# Transcriptomic and proteomic analysis reveals mechanisms of low pollen-pistil compatibility during water lily cross breeding

**DOI:** 10.1186/s12870-019-2166-3

**Published:** 2019-12-05

**Authors:** Chun-Qing Sun, Fa-Di Chen, Nian-Jun Teng, Yue-Mei Yao, Xi Shan, Zhong-Liang Dai

**Affiliations:** 1 Zhenjiang Institute of Agricultural Science in Jiangsu Hilly Areas, Jurong, 212400 China; 2 College of Horticulture, Nanjing Agricultural University, Nanjing, 210095 China

**Keywords:** *Nymphaea*, Interspecific reproductive barriers, Transcriptomic, Proteomic, Pollen-stigma interaction, ROS, Flavonoids

## Abstract

**Background:**

In water lily (*Nymphaea*) hybrid breeding, breeders often encounter non-viable seeds, which make it difficult to transfer desired or targeted genes of different *Nymphaea* germplasm. We found that pre-fertilization barriers were the main factor in the failure of the hybridization of *Nymphaea*. The mechanism of low compatibility between the pollen and stigma remains unclear; therefore, we studied the differences of stigma transcripts and proteomes at 0, 2, and 6 h after pollination (HAP). Moreover, some regulatory genes and functional proteins that may cause low pollen-pistil compatibility in *Nymphaea* were identified.

**Results:**

RNA-seq was performed for three comparisons (2 vs 0 HAP, 6 vs 2 HAP, 6 vs 0 HAP), and the number of differentially expressed genes (DEGs) was 8789 (4680 were up-regulated), 6401 (3020 were up-regulated), and 11,284 (6148 were up-regulated), respectively. Using label-free analysis, 75 (2 vs 0 HAP) proteins (43 increased and 32 decreased), nine (6 vs 2 HAP) proteins (three increased and six decreased), and 90 (6 vs 0 HAP) proteins (52 increased and 38 decreased) were defined as differentially expressed proteins (DEPs). Gene Ontology (GO) and Kyoto Encyclopedia of Genes and Genomes (KEGG) enrichment analyses revealed that the DEGs and DEPs were mainly involved in cell wall organization or biogenesis, S-adenosylmethionine (SAM) metabolism, hydrogen peroxide decomposition and metabolism, reactive oxygen species (ROS) metabolism, secondary metabolism, secondary metabolite biosynthesis, and phenylpropanoid biosynthesis.

**Conclusions:**

Our transcriptomic and proteomic analysis highlighted specific genes, incuding those in ROS metabolism, biosynthesis of flavonoids, SAM metabolism, cell wall organization or biogenesis and phenylpropanoid biosynthesis that warrant further study in investigations of the pollen-stigma interaction of water lily. This study strengthens our understanding of the mechanism of low pollen-pistil compatibility in *Nymphaea* at the molecular level, and provides a theoretical basis for overcoming the pre-fertilization barriers in *Nymphaea* in the future.

## Background

Water lilies (*Nymphaea*) are important flowering plants that are distributed worldwide from the tropics to temperate regions [1]. With the rapid improvement of China’s economy and the overall quality of life, the demand is increasing for new water lily hybrids with different characteristics. Therefore, it is necessary to breed new water lily hybrids with excellent ornamental characteristics. However, in the breeding of water lily hybrids, breeders often encounter non-viable seeds, which makes it difficult to transfer the desired or targeted genes of various water lily germplasm [2, 3]. For example, many breeders have hoped to transfer the colorful flowers of tropical water lilies to hardy water lilies through crossbreeding; however, viable hardy water lily varieties with blue flowers have not yet been developed [4]. In recent years, we have carried out interspecific hybridization between the female *N*. ‘Peter Slocum’ and male *N. micrantha* for three consecutive years, aiming at transferring the color gene of male parent to female parent. However, we also did not obtain seeds, so we carried out a thorough and systematic study from the aspect of plant reproductive biology, and found that the main reason for the failure of the hybrid combination was the low compatibility between pollen and stigma before fertilization [3]. Therefore, in this study, an interspecific cross between the female ‘Peter Slocum’ and male *N. micrantha* was performed. Our aim was to further reveal the reasons of low compatibility between pollen and stigma at the molecular level on the basis of previous studies.

Low compatibility between the pollen and stigma is a common issue that negatively impacts the efficiency of plant breeding and the yield of seeds or fruit [5, 6]. Therefore, over the past several decades, many researchers have conducted studies to investigate factors that cause low compatibility between the pollen and stigma [7–10]. However, the mechanisms underlying low compatibility between the pollen and stigma in *Nymphaea* remain poorly understood.

With the development of molecular biology technology, the use of transcriptome and proteomics technology may provide a new way to find the genes and proteins related to low compatibility between pollen and stigma [11–13]. In particular, transcriptome sequencing is a useful method for identifying novel transcripts and analyzing gene expression [14, 15]. Transcriptomic and proteomic analyses have been extensively applied to many plant species, but limited transcriptome and proteome data exists regarding pre-fertilization barriers in water lily [16, 17]. To understand the mechanism of low pollen-pistil compatibility in water lily at the genomic level, Illumina paired-end sequencing and a label-free analysis of the stigma after pollination were conducted. This comprehensive analysis of the transcriptome and proteome may substantially improve the overall understanding of the potential molecular mechanisms involved in low pollen-pistil compatibility in water lily and pave the way for further analyses. This study aimed to provide important molecular data supporting a deep understanding of low compatibility between the pollen and stigma in water lily and also provides an important clue to overcome hybridization barriers.

## Results

### Pollen germination on stigmas after pollination

Previous studies showed that pollen began to germinate at 2 HAP, and abnormal growth of pollen tubes was observed at 6 HAP [3]. The number of pollen tubes did not change at 6 and 12 HAP, indicating that the related genes in stigma had been expressed and new proteins were synthesized, while no new proteins were synthesized after 6 h. For this reason, the stigmas of 0, 2, and 6 HAP were collected.

**Fig. 1:**
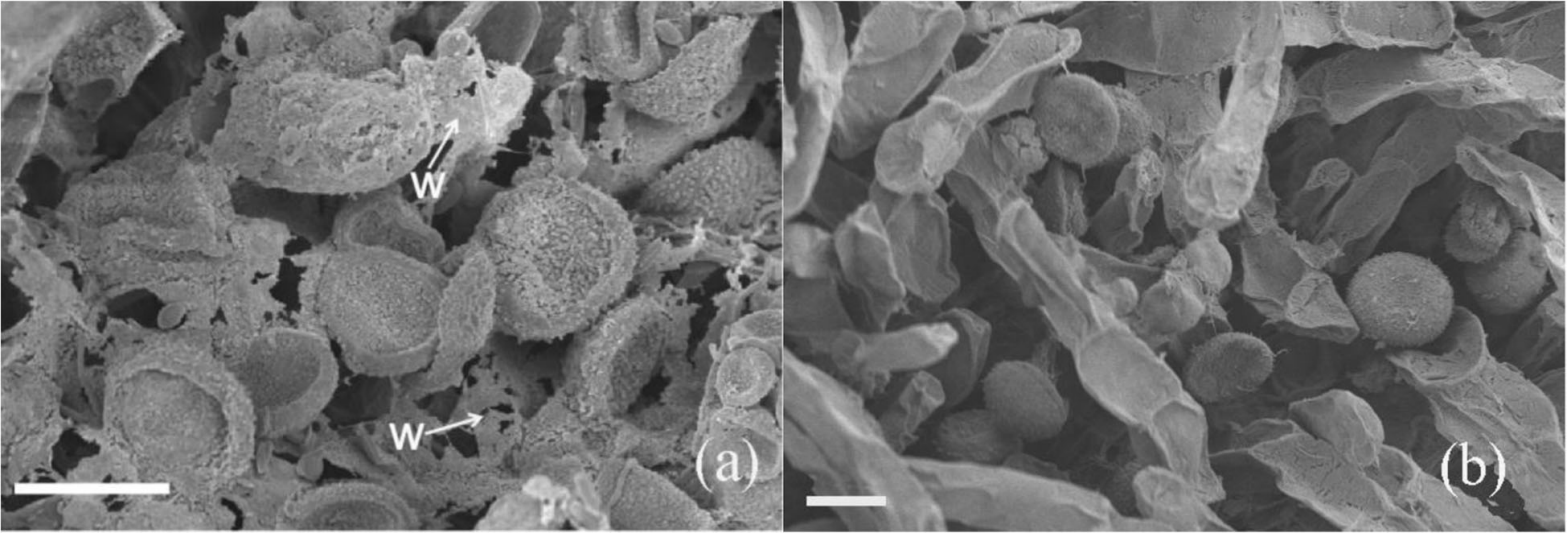
Pollen germination on stigmas at 6 h after pollination with scanning electron microscope. **a** In the ‘Peter Slocum’ × *N. micrantha* cross, accumulation of wax between the stigma and the surface of the pollen grains was commonly observed. **b** To the self-pollinated ‘Peter Slocum’, the stigma and the surface of the pollen grains showed no wax. Scale bar = 25 μm. W= Wax

In the ‘Peter Slocum’ × *N. micrantha* cross, no pollen tubes penetrated stigmatic tissue between 2 and 6 HAP. In addition, the accumulation of wax between the stigma and the surface of the pollen grains was commonly observed (Fig. 1a). In the self-pollinated ‘Peter Slocum’, the stigma and the surface of the pollen grains showed no wax at 6 HAP (Fig. 1b).

### Overview of the transcriptomic analysis and proteomics analysis

Using Fragments Per Kilobase Million (FPKM), we explored the gene expression levels in the stigmas 0, 2, and 6 HAP. In three comparisons (2 vs 0 HAP, 6 vs 2 HAP, 6 vs 0 HAP), the number of DEGs was 8789 (4680 were up-regulated), 6401 (3020 were up-regulated), and 11,284 (6148 were up-regulated), respectively. Further details of the DEGs are presented in Additional file 1. Using the label-free analysis, a total of 3176 proteins were identified within a false discover rate (FDR) of 1% (Additional file 2). Following the proteins (2 vs 0 HAP; 43 increased and 32 decreased), nine proteins (6 vs 2 HAP; three increased and six decreased), and 90 proteins (6 vs 0 HAP; 52 increased and 38 decreased) were defined as DEPs (Additional file 3).

### Comparison analysis of transcriptome and proteome data

**Fig. 2:**
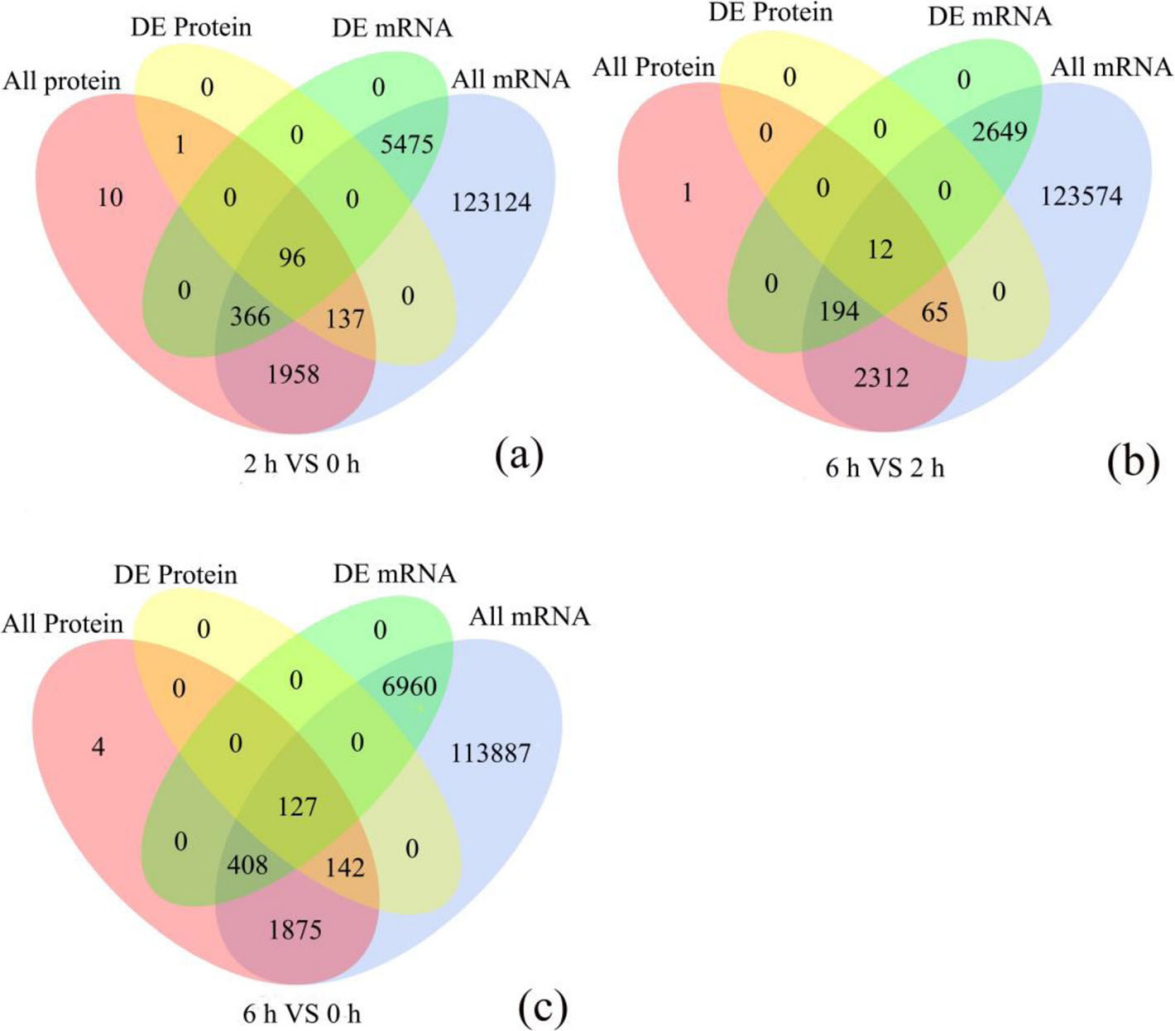
Venn diagram showing all identified, as well as all significantly enriched, mRNAs and proteins and their overlap. All protein: Represents all quantifiable proteins in the proteome; All Gene: Represents all quantifiable genes obtained in the transcriptome; DE Protein: Represents differentially expressed proteins identified by the proteome; DE mRNA: Represents differentially expressed genes identified by the transcriptome

To identify robust pathways that were supported by both datasets, we integrated DEGs and DEPs to find the corresponding genes and proteins, and the results are listed in Additional file 4. Overlaps between DEPs and DEGs are shown by Venn diagrams in Fig. 2. Specifically, there were considerable non-overlaps between DEPs and DEGs, probably due to the relatively low sensitivity of proteome detection. For instance, among the 234 differentially regulated proteins in the 2 vs 0 HAP comparison, only 96 genes and their corresponding proteins were differentially expressed (Fig 2a). Similarly, for the 6 vs 2 HAP and 6 vs 0 HAP comparisons, 12 and 127 of the DEPs, respectively, were correlated to the corresponding DEGs (Fig. 2b, c).

**Fig. 3:**
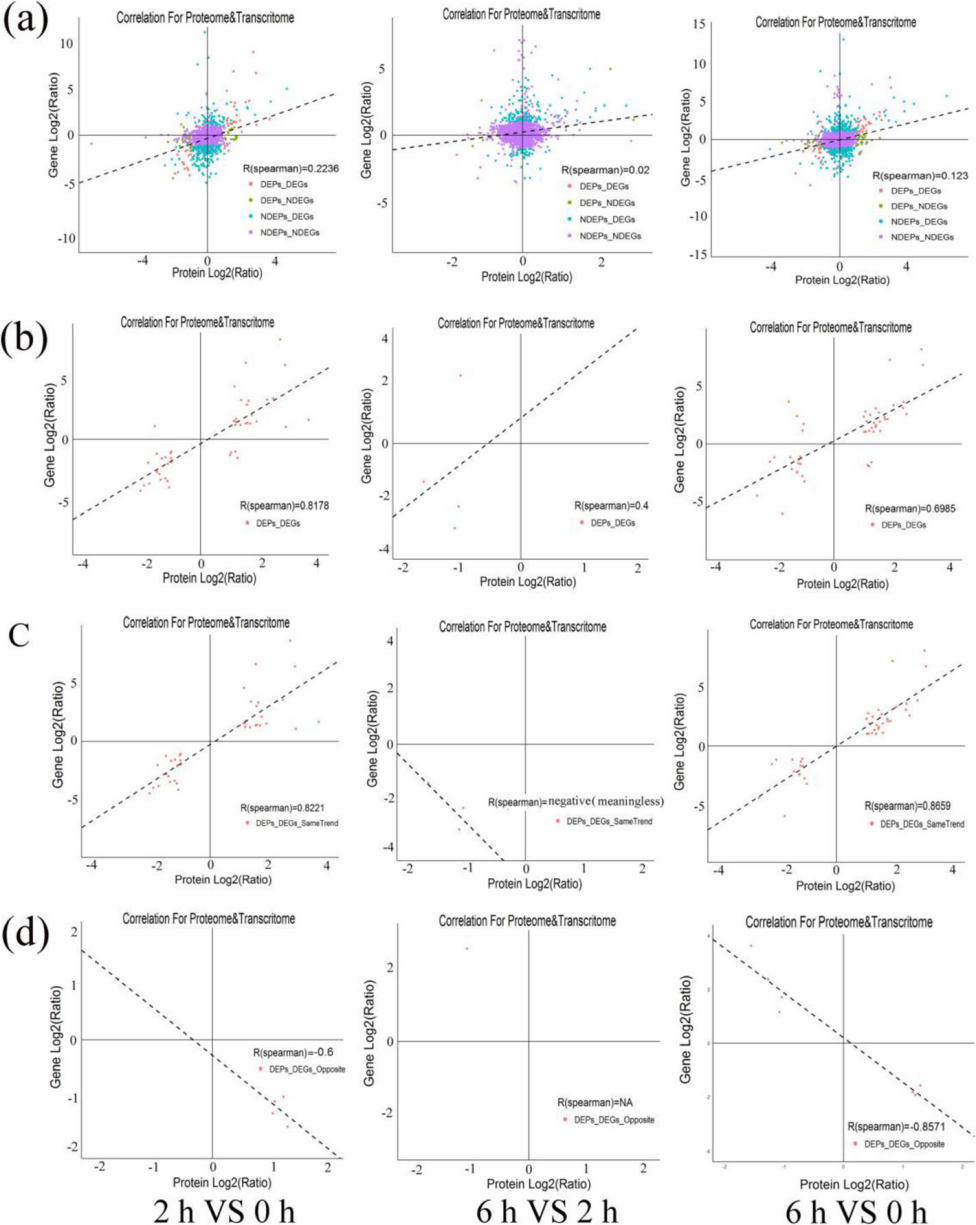
Correlations between protein and messenger ribonucleic acid (mRNA) expression. x-axis represents the protein expression level, and y-axis represents the genes expression level. **a** Scatterplots of the relationship between genes quantified in both transcriptomic and proteomic data sets. **b** Scatterplots and correlation coefficients between differentially expressed proteins (DEPs) and differently expressed genes (DEGs). Scatterplots and correlation coefficients between proteins and mRNA expression ratios which are the same (**c**) or opposite (**d**) changing tendency. The purple plot indicates none DEPs and DEGs; green plot indicates DEPs but none DEGs; blue plot indicates DEGs but none DEPs; red plot indicates DEPs and DEGs, and all data were log2-transformed

All expression data associated with protein level and transcription level were analyzed and Spearman correlation coefficient was calculated. Globally, the correlation coefficients of all quantitative proteins and their corresponding genes at 2 vs 0 HAP, 6 vs 2 HAP, and 6 vs 0 HAP were 0.2236, 0.02 and 0.123, respectively (Fig. 3a). However, there is a high correlation between the DEGs and their corresponding DEPs (*r* = 0.8178, 0.4, and 0.6985, respectively; Fig. 3b). The correlation between proteins and their corresponding mRNAs with the same or opposite trend was analysed, and the comparative group 2 vs 0 HAP and 6 vs 0 HAP had higher positive or negative correlations (Fig. 3c and Fig. 3d). However, we found poor correlations between proteins at 6 vs 2 HAP and their corresponding mRNAs with the same or opposite trend (Fig. 3c and Fig. 3d). Among the Correlation-DEGs-DEPs (cor-DEGs-DEPs) genes, 39 (2 vs 0 HAP), three (6 vs 2 HAP), and 41 (6 vs 0 HAP) genes had the same trend, while five (2 vs 0 HAP), one (6 vs 2 HAP), and seven (6 vs 0 HAP) genes had the opposite trend (Additional file 5). Thus, we suggest that some of these cor-DEGs-DEPs genes might play important roles in causing low pollen-pistil compatibility during water lily breeding.

### Cluster analysis of expression patterns in the cor-DEGs-DEPs genes

**Fig. 4:**
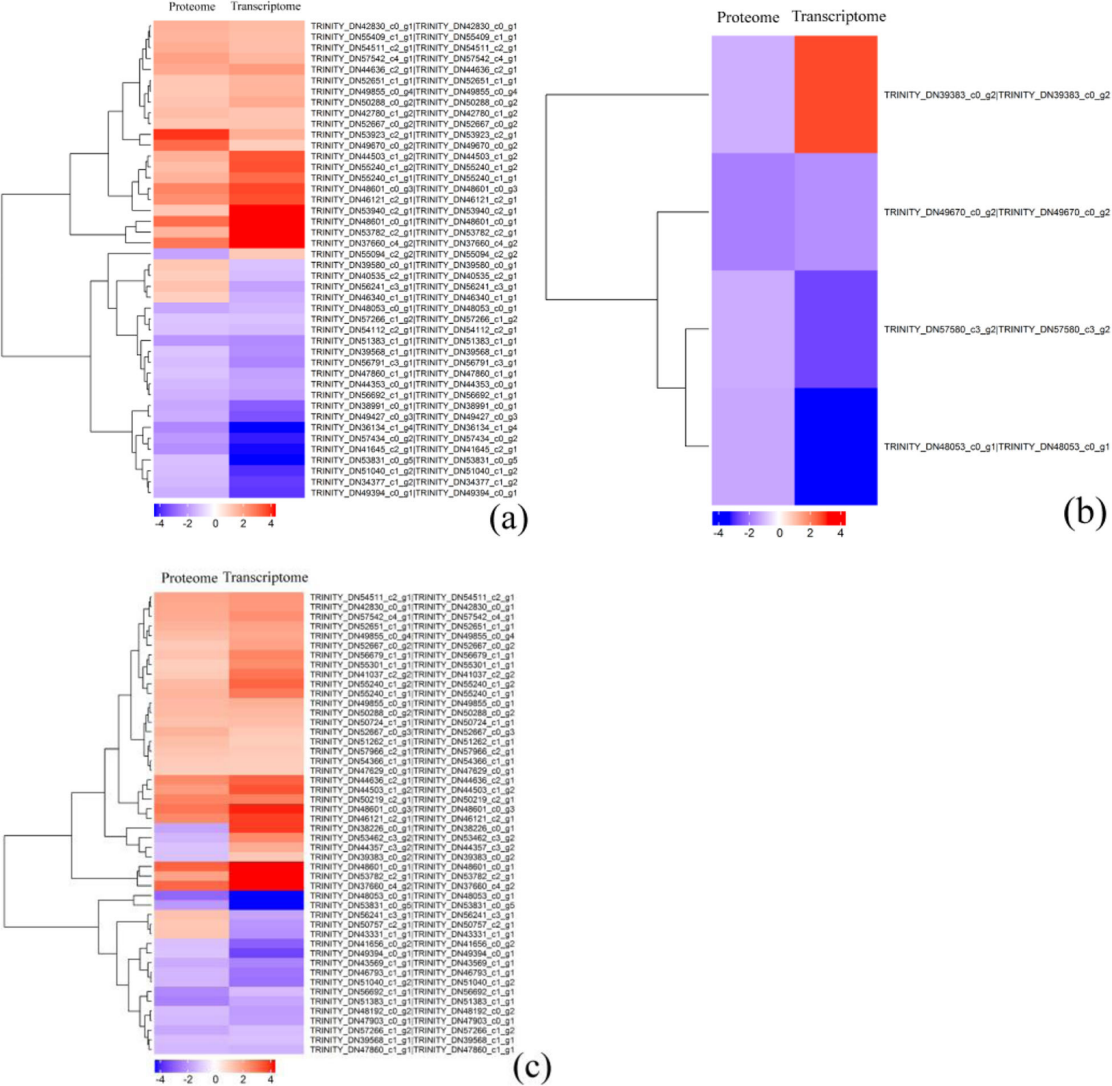
Cluster analysis of associated differential proteins and differential mRNA expression patterns. **a** 2 h vs 0 h. (**b**) 6 h vs 2 h. **c** 6 h vs 0 h. Each row in the graph represents a protein/mRNA, and each column in the graph represents a sample (the proteome sample on the left and the corresponding transcriptome sample on the right). Numbers are listed as the log 2 value of difference multiples. Expression differences are shown in different colors; red indicates up-regulation, while blue indicates down-regulation.

Cluster analysis of the DEPs and their corresponding DEGs can visually show their expression patterns, and the results are shown in Fig. 4. Cluster analysis showed that 44 (2 vs 0 HAP), four (6 vs 2 HAP), and 48 (6 vs 0 HAP) DEPs were correlated with the change of mRNA abundance, and 39 (2 vs 0 HAP), three (6 vs 2 HAP), and 41 (6 vs 0 HAP) DEPs were matched with corresponding DEGS. However, 5 (2 vs 0 HAP), one (6 vs 2 HAP) and seven (6 vs 0 HAP) DEPS were opposite to their mRNA expression pattern. In total, 25 of the cor-DEGs-DEPs genes at 2 vs 0 HAP and 6 vs 0 HAP showed the same expression pattern; thus, we infer that these genes may cause low pollen-pistil compatibility in interspecific hybridization of water lily.

### GO and pathway enrichment analysis of the cor-DEGs-DEPs genes

**Fig. 5:**
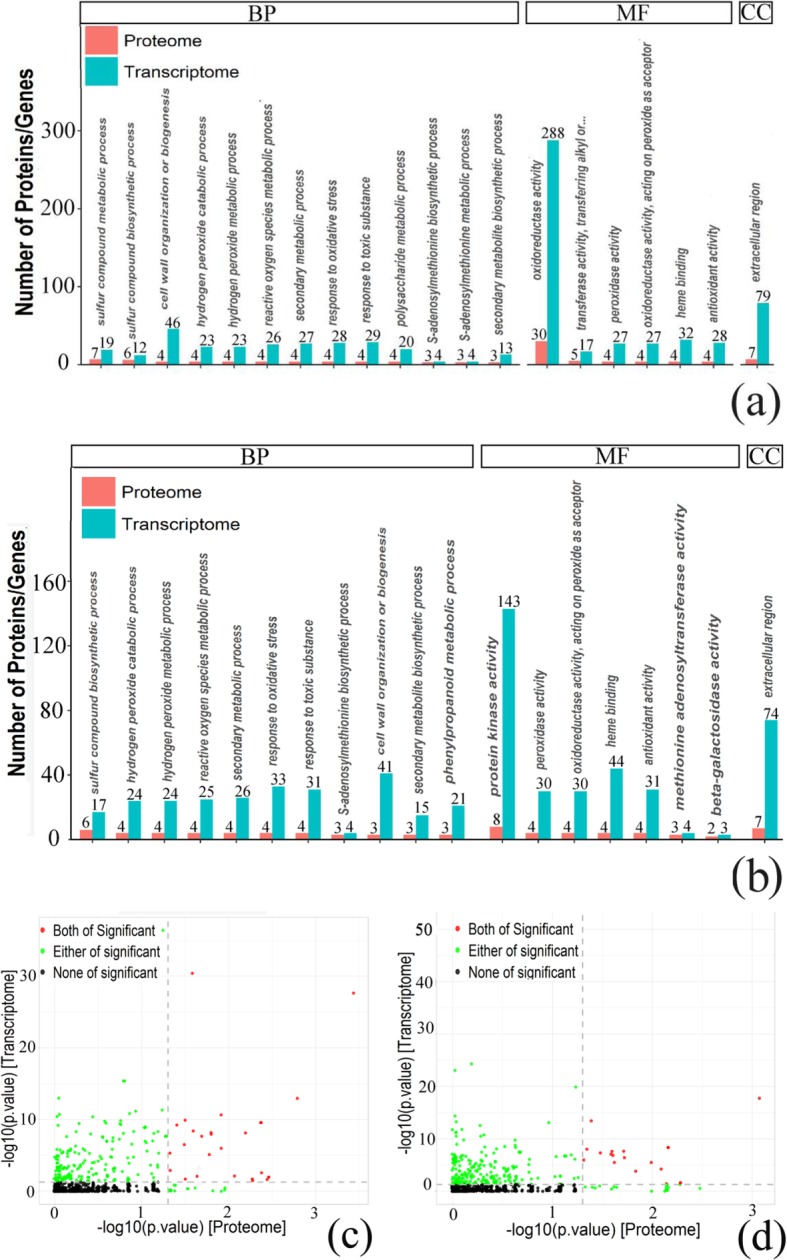
GO enrichment analyses of DEGs and DEPs. **a**, **c** 2 h vs 0 h. **b**, **d** 6 h vs 0 h. **a**, **b** Number of GO enrichment correlation between proteome and transcriptome. Each column in the figure represents a GO secondary annotation entry, red represents differentially expressed proteins, and blue represents differentially expressed genes. From left to right, the number of differentially expressed proteins is arranged from high to low. BP: Biological process, CC: Cellular component, MF: Molecular function. **c**, **d** The overview scatter diagram of GO enrichment correlation between the protein level and transcript level of genes

After obtaining the expression information of the cor-DEGs-DEPs genes at 2 vs 0 HAP, 6 vs 2 HAP, and 6 vs 0 HAP, GO functional annotation analysis of these genes was carried out (Table 1; Table 2). The results showed that 27 (2 vs 0 HAP), 0 (6 vs 2 HAP), 19 (6 vs 0 HAP) GO terms were highly enriched at both mRNA and protein levels (Fig. 5; Additional file 6). The subcategory identified in the cellular component category was extracellular region in both 2 vs 0 HAP and 6 vs 0 HAP. For the molecular function category, peroxidase activity, heme binding, antioxidant activity, and oxidoreductase activity, acting on peroxide as acceptor were the most abundant categories in both 2 vs 0 HAP and 6 vs 0 HAP. The most abundant biological processes categories identified in both 2 vs 0 HAP and 6 vs 0 HAP were cell wall organization or biogenesis, phenylpropanoid metabolic process, sulfur compound biosynthetic process, hydrogen peroxide catabolic process, and ROS metabolic process. In addition, no GO terms were significantly enriched in the cor-DEGs-DEPs genes at 6 vs 2 HAP.

**Table 1:** Correlated diferentially expression transcripts/proteins for the comparison between 2 HAP and 0 HAP

Correlation ID	Description	GO Annotation or KEEG pathway	Expression pattern
TRINITY_DN39580_c0_g1	Putative xyloglucan endotransglucosylase/hydrolase [*Ananas comosus*]	Cell wall organization or biogenesis	+(−)
TRINITY_DN47677_c0_g1	Hypothetical protein AMTR_s00019p00242980 [*Amborella trichopoda*]	Cell wall organization or biogenesis	+(+)
TRINITY_DN39580_c0_g1	Probable xyloglucan endotransglucosylase/hydrolase [*Ananas comosus*]	Cell wall organization or biogenesis	+(−)
TRINITY_DN51485_c1_g3	Predicted: omega-hydroxypalmitate O-feruloyl transferase-like [*Nelumbo nucifera*]	Cutin, suberine and wax biosynthesis	+(+)
TRINITY_DN52854_c0_g1	Beta-galactosidase [*Amborella trichopoda*]	Galactosidase activity	+(+)
TRINITY_DN50844_c2_g2	Beta-galactosidase-like isoform X2 [*Quercus suber*]	Galactosidase activity	+(−)
TRINITY_DN53009_c1_g1	Endoglucanase 8 isoform X1 [*Amborella trichopoda*]	Polysaccharide metabolic process	-(−)
TRINITY_DN46121_c2_g1	O-methyltransferase [*Macleaya cordata*]	Flavonoid biosynthesis	+(+)
TRINITY_DN55094_c2_g2	Flavonol synthase [*Allium cepa*]	Flavonoid biosynthesis	-(+)
TRINITY_DN44636_c2_g1	Predicted: UDP-glycosyltransferase 88B1-like [*Nelumbo nucifera*]	Flavonoid biosynthesis	+(+)
TRINITY_DN40390_c1_g3	Predicted: S-adenosylmethionine synthase 1 [*Nelumbo nucifera*]	S-adenosylmethionine biosynthetic process	+(+)
TRINITY_DN52955_c1_g5	S-adenosylmethionine synthase 1 [*Pinus pinaster*]	S-adenosylmethionine biosynthetic process	+(+)
TRINITY_DN52955_c1_g5	S-adenosylmethionine synthase 1 [*Pinus pinaster*]	S-adenosylmethionine biosynthetic process	+(+)
TRINITY_DN48053_c0_g1	Thiamine thiazole synthase, chloroplastic-like [*Lactuca sativa*]	Sulfur compound biosynthetic process	-(−)
TRINITY_DN46858_c0_g1	Mitogen-activated protein kinase 6 [*Pinus massoniana*]	Sulfur compound biosynthetic process	+(+)
TRINITY_DN49886_c0_g4	Predicted: glutathione S-transferase [*Cucumis sativus*]	Sulfur compound metabolic process	+(−)
TRINITY_DN43428_c2_g1	Phenylalanine ammonia lyase, partial [*Tulipa fosteriana*]	Phenylpropanoid metabolic process	+(+)
TRINITY_DN43428_c2_g2	Predicted: phenylalanine ammonia-lyase [*Vitis vinifera*]	Phenylpropanoid metabolic process	+(+)
TRINITY_DN53782_c2_g1	Predicted: peroxidase 72-like [*Phoenix dactylifera*]	Phenylpropanoid biosynthesis	+(+)
TRINITY_DN35350_c0_g1	Cytochrome P450 98A2 [*Cajanus cajan*]	Phenylpropanoid metabolic process	+(+)
TRINITY_DN37660_c4_g2	Predicted: peroxidase 4-like [*Nelumbo nucifera*]	Hydrogen peroxide catabolic process	+(+)
TRINITY_DN38816_c2_g1	Hypothetical protein POPTR_0013s08130g [*Populus trichocarpa*]	Hydrogen peroxide catabolic process	+(+)
TRINITY_DN50288_c0_g2	F5 M15.5 [*Arabidopsis thaliana*]	Peroxidase activity	+(+)
TRINITY_DN36305_c6_g1	Predicted: laccase-15-like [*Nelumbo nucifera*]	Oxidoreductase activity	+(+)
TRINITY_DN37177_c0_g1	Uncharacterized protein LOC18047357 isoform X6 [*Citrus clementina*]	Oxidoreductase activity	+(−)
TRINITY_DN39968_c1_g2	Aldehyde dehydrogenase family 3 member h1-like protein, partial [*Trifolium pratense*]	Oxidoreductase activity	+(+)
TRINITY_DN44353_c0_g1	Predicted: alcohol dehydrogenase 1 [*Elaeis guineensis*]	Oxidoreductase activity	-(−)
TRINITY_DN47125_c2_g3	Geraniol 8-hydroxylase [*Amborella trichopoda*]	Oxidoreductase activity	-(−)
TRINITY_DN48601_c0_g1	Predicted:probable NAD (P) H dehydrogenase (quinone) FQR1-like 1 [*Tarenaya hassleriana*]	Oxidoreductase activity	+(+)
TRINITY_DN48601_c0_g3	Predicted: probable NAD (P) H dehydrogenase (quinone) FQR1-like 1 [*Ipomoea nil*]	Oxidoreductase activity	+(+)
TRINITY_DN49379_c0_g1	Hypothetical protein AQUCO_02400018v1 [*Aquilegia coerulea*]	Oxidoreductase activity	+(+)
TRINITY_DN49670_c0_g2	Ubiquinol oxidase [*Handroanthus impetiginosus*]	Oxidoreductase activity	+(+)
TRINITY_DN51072_c1_g3	Hypothetical protein PHAVU_009G140700g [*Phaseolus vulgaris*]	Oxidoreductase activity	+(+)
TRINITY_DN52147_c0_g1	Predicted: peroxidase 17-like [*Nelumbo nucifera*]	Oxidoreductase activity	+(+)
TRINITY_DN56692_c1_g1	Predicted: geraniol 8-hydroxylase-like [*Elaeis guineensis*]	Oxidoreductase activity	-(+)
TRINITY_DN58079_c0_g1	Predicted: putative laccase-9 [*Elaeis guineensis*]	Oxidoreductase activity	+(+)

Note: “+” or “−” outside the brackets indicates that the expression at 2 HAP is up- or down-regulated than at 0 HAP at the proteomic level; “+” or “−” inside the brackets indicates that the expression at 2 HAP is up- or down-regulated than at 0 HAP at the transcriptome level

**Table 2:** Correlated diferentially expression transcripts/proteins for the comparison between 6 HAP and 0 HAP

Correlation ID	Description	GO annotation or KEEG pathway	Expression pattern
TRINITY_DN37660_c4_g2	Predicted: peroxidase 4-like [*Nelumbo nucifera*]	Hydrogen peroxide catabolic process	+(+)
TRINITY_DN38816_c2_g1	Hypothetical protein POPTR_0013s08130g [*Populus trichocarpa*]	Hydrogen peroxide catabolic process	+(+)
TRINITY_DN50202_c1_g1	Cationic peroxidase 1 [*Amborella trichopoda*]	Hydrogen peroxide catabolic process	+(+)
TRINITY_DN50288_c0_g2	F5 M15.5 [*Arabidopsis thaliana*]	Hydrogen peroxide catabolic process	+(+)
TRINITY_DN40390_c1_g3	Predicted: S-adenosylmethionine synthase 1 [*Nelumbo nucifera*]	S-adenosylmethionine biosynthetic process	+(+)
TRINITY_DN52955_c1_g5	S-adenosylmethionine synthase 1 [*Pinus pinaster*]	S-adenosylmethionine biosynthetic process	+(+)
TRINITY_DN47677_c0_g1	Hypothetical protein AMTR_s00019p00242980 [*Amborella trichopoda*]	Cell wall organization or biogenesis	+(+)
TRINITY_DN50844_c2_g2	Beta-galactosidase-like isoform X2 [*Quercus suber*]	Galactosidase activity	+(−)
TRINITY_DN52854_c0_g1	Beta-galactosidase [*Amborella trichopoda*]	Galactosidase activity	+(+)
TRINITY_DN46858_c0_g1	Mitogen-activated protein kinase 6 [*Pinus massoniana*]	Sulfur compound biosynthetic process	+(+)
TRINITY_DN48053_c0_g1	Thiamine thiazole synthase, chloroplastic-like [*Lactuca sativa*]	Sulfur compound biosynthetic process	-(−)
TRINITY_DN35350_c0_g1	Cytochrome P450 98A2 [*Cajanus cajan*]	Phenylpropanoid metabolic process	+(+)
TRINITY_DN43428_c2_g1	Phenylalanine ammonia lyase, partial [*Tulipa fosteriana*]	Phenylpropanoid metabolic process	+(+)
TRINITY_DN43428_c2_g2	Predicted: phenylalanine ammonia-lyase [*Vitis vinifera*]	Phenylpropanoid metabolic process	+(+)
TRINITY_DN53782_c2_g1	Predicted: peroxidase 72-like [*Phoenix dactylifera*]	Phenylpropanoid biosynthesis	+(+)
TRINITY_DN50219_c2_g1	4-coumarate-CoA ligase [*Cinnamomum osmophloeum*]	Phenylpropanoid biosynthesis	+(+)
TRINITY_DN46121_c2_g1	O-methyltransferase [*Macleaya cordata*]	Flavonoid biosynthesis	+(+)

Note: “+” or “−” outside the brackets indicates that the expression at 2 HAP is up- or down-regulated than at 0 HAP at the proteomic level; “+” or “−” inside the brackets indicates that the expression at 2 HAP is up- or down-regulated than at 0 HAP at the transcriptome level

**Fig. 6:**
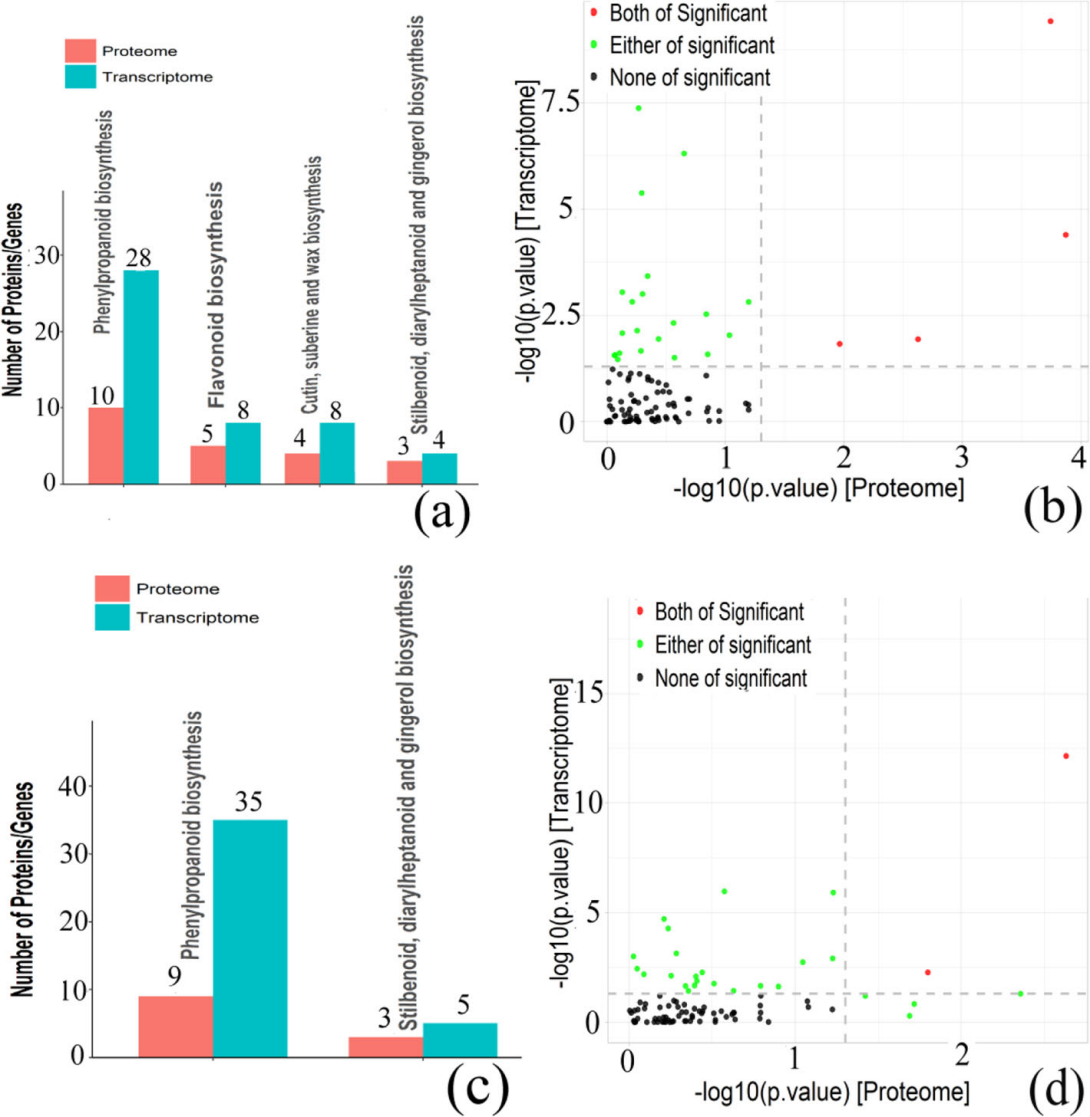
KEGG enrichment analyses of DEGs and DEPs. **a**, **b** 2 h vs 0 h. **c**, **d** 6 h vs 0 h. **a**, **c** Number of KEGG enrichment correlation between proteome and transcriptome. Each column in the figure represents a KEGG pathway, and different colors represent different histology. The red column in the figure represents the KEGG enrichment result of proteome, and the blue column represents the KEGG enrichment result of transcriptome. The abscissa is the name of the enriched KEGG pathway, and the ordinate represents the number of enriched proteomes and transcriptomes. From left to right, the number of differentially expressed proteins ranged from high to low. **b**, **d** The overview scatter diagram of KEGG enrichment correlation between the protein level and transcript level of genes

KEGG pathway analysis can help us further understand the biochemical metabolic pathways and signal transduction pathways involved in the cor-DEGs-DEPs genes (Fig. 6; Table 1; Table 2; Additional file 7). The results showed that two KEGG pathways were highly enriched at both mRNA and protein levels including phenylpropanoid biosynthesis (ko00940) and stilbenoid, diarylheptanoid, and gingerol biosynthesis (ko00945) in both 2 vs 0 HAP and 6 vs 0 HAP. For the cor-DEGs-DEPs genes at 2 vs 0 HAP, cutin, suberin, and wax biosynthesis (ko00073) and flavonoid biosynthesis (ko00941) were significant pathways in both the proteome and transcriptome. In addition, no KEGG pathways were significantly enriched in the cor-DEGs-DEPs genes at 6 vs 2 HAP.

### Parallel reaction monitoring (PRM) analysis

Four differentially expressed proteins (mainly related to flavonoid biosynthesis, peroxidase activity and phenylpropanoid biosynthesis) were chosen for PRM analysis. According to the relative expression quantity of the corresponding peptide fragment of four target proteins in peptide mixtures of the stigmas of 0, 2, and 6 HAP, the relative expression quantity differences of target proteins were obtained (Table 3). Detailed protein quantitative information and significant difference analysis results are shown in Additional file 8. The results from this analysis indicated that expression quantities of the four target proteins in the 2 vs 0 HAP and 6 vs 0 HAP comparisons were markedly up-regulated, whereas the expression quantity of the four target proteins in the 6 vs 2 HAP comparison was not significantly changed. The results of the PRM analysis indicated that the four candidate proteins show similar trends as the label-free results, which supported the credibility of the proteomics data.

**Table 3:** Relative quantitative of target peptide segment by PRM analysis

Protein Name	Ratio 2 HAP/0 HAP	Ratio 6 HAP/0 HAP	Ratio 6 HAP/2 HAP	TTEST 2 HAP/0 HAP	TTEST 6 HAP/0 HAP	TTEST6 HAP/2 HAP
TRINITY DN44636 c2 g1	2.56	3.38	1.32	0.04814	0.00136	0.24484
TRINITY DN50288 c0 g2	3.33	4.19	1.26	0.04013	0.00353	0.38647
TRINITY DN35350 c0 g1	5.06	5.98	1.18	0.00734	0.00105	0.40440
TRINITY DN53782 c2 g1	3.03	4.01	1.32	0.00635	0.00137	0.13851

## Discussion

### Low pollen-pistil compatibility during water lily breeding are associated with the metabolism of ROS

In this study, the combined transcriptome and proteome analysis showed that the expression of genes and proteins related to the metabolism of ROS on the stigma increased significantly in the 2 vs 0 HAP and 6 vs 0 HAP comparisons, suggesting that ROS may be involved in regulating the interaction between the pollen and stigma of water lily after pollination. ROS participate in many pollen-related processes, such as tapetum and pollen development [18–20], in vitro pollen germination [21], growth of the pollen tube apex [22–24], the rupture of the pollen tube to release sperm [25], and self-incompatibility [26]. The role of ROS in pollen tube growth has been well established, but little is known about its involvement in the early stage of pollen germination. The biological function of ROS and hydrogen peroxide on the stigma may be involved in some signal crosslinks in the interaction between the pollen and stigma [27, 28]. Numerous experiments have shown that mature pollen grains produce a large amount of Nitric Oxide (NO), which inhibits ROS production in stigma papilla cells [27, 29, 30]. The mutual exclusion of ROS and NO during pollen tube growth suggests that there may be a coordination mechanism between these signaling molecules during pollen tube growth [31]; this which indicates that ROS from the stigma and NO from the pollen participate in the pollen-stigma interaction as signaling molecules [32]. In addition, ROS are mainly composed of hydrogen peroxide on the stigma, which is considered the most important redox signaling molecule because of its unique physical and chemical properties as well as its stability in cells. Hydrogen peroxide can oxidize the thiol group of target protein cysteine, thus changing the structure and function of proteins [33].

However, the regulatory mechanism of ROS in the interaction between pollen and stigma of water lily after pollination is unclear. We infer that low pollen-pistil compatibility after pollination is due to the change of the level of ROS on the stigma. ROS may act as a signaling molecule to oxidize downstream target proteins. The oxidized target proteins cannot function properly, affecting the pollen germination on the stigma surface. Therefore, the metabolic process of ROS on the stigma may be related to the interaction between the pollen and the stigma of water lily.

### Effects of stigma flavonoids on low pollen-pistil compatibility during water lily breeding

Flavonoids, which can affect plant physiology, growth, and development, are common secondary metabolites in vascular plants [34, 35]. Flavonoids are mainly involved in the physiological processes of plant symbiosis, defense against disease and insect pests, auxin transport, seed and pollen germination, and root development. Flavonoids are needed for pollen germination and stimulate pollen tube growth in some plant species [36, 37]. Using pollen produced by in vitro culture of immature pollen of tobacco (*Nicotiana tabacum* L.) and petunia (*Petunia hybrida*) as materials, adding flavonol (quercetin, kaempferol, myricetin) to germination medium could significantly promote pollen germination frequency and pollen tube growth in vitro [36]. Similarly, the pollen of a flavonoid-deficient mutant of petunia could not germinate on the stigma, but the addition of the exogenous flavonoid kaempferol could induce pollen germination on the stigma. This indicates that kaempferol may play a role in pollen germination [37]. In this study, our transcriptome and proteome analyses showed that there were significant differences in genes and proteins related to flavonoid biosynthesis between stigmas at 0 HAP and stigmas at 2 and 6 HAP. For example, the expression and content of flavonol synthase in stigmas after pollination are lower than those of unpollinated stigmas, which indicates that the flavonoid content on the stigma after pollination is greatly reduced. In addition, flavonoids can affect plant reproductive and developmental processes and participate in the interaction between the pollen and stigma [38, 39]. Other transcriptomic studies on rice stigmas revealed that numerous genes encoding flavonols were expressed, and these genes were expressed on rice stigmas, suggesting that flavonoids play an important role in the interaction between pollen and stigma [38]. Therefore, we infer that the biosynthesis of flavonoids is closely related to the low pollen-pistil compatibility during water lily breeding.

### Effects of SAM metabolism on low pollen-pistil compatibility during water lily breeding

The SAM participates in many important physiological processes, such as transamination of propyl, methyl, and sulfur in plants, and is the main hub of methionine metabolism [40, 41]. The results in the present study showed that the SAM synthase gene was up-regulated by pollination. SAM can also be used as a precursor of ethylene and polyamine [42]. Moreover, polyamines with appropriate concentrations are important for pollen germination and pollen tube growth [43]. Similarly, a previous study has shown that ethylene plays a role in pollen tube growth after pollination in tobacco [44]. We infer that up-regulation of genes related to S-adenosylmethionine synthesis and metabolism induced by pollination may facilitate the synthesis of polyamines and ethylene by the stigma papilla cells, and then regulate pollen germination and pollen tube growth. Thus, it is speculated that the metabolic pathway of SAM participates in the interaction between the pollen and stigma of water lily and plays an important role in the regulation mechanism of the pollen-stigma interaction.

### Cell wall organization or biogenesis is associated with low pollen-pistil compatibility during water lily breeding

During the interaction between the pollen and stigma, some enzymes in stigma papilla cells are activated and released by certain signals. These enzymes are mainly involved in modifying cell walls, such as enzymes that degrade pectin, cellulose, and hemicellulose [45–47]. In our study, the combined transcriptome and proteome analysis showed that many DEGs involved in cell wall tissue metabolism were found on stigma at 2 and 6 h after pollination, suggesting that genes involved in cell wall synthesis were induced by pollination. Transcriptome studies on the stigma of many species have confirmed that there are a large number of cell wall metabolism-related genes in the stigma [48–50], and the products of these genes may be secreted by stigma papillae to help pollen tubes penetrate the stigma. In addition, the GO annotation showed that the expression of endoglucanase involved in cellulose hydrolysis was significantly down-regulated, while beta-galactosidase and xyloglucan endotransglucosylase/hydrolase genes involved in cell wall modification were significantly up-regulated. Thus, we infer that beta-galactosidase may be involved in cellulose synthesis and cell wall elongation during cell wall metabolism of *Nymphaea* stigmas [51], whereas xyloglucan endotransglucosylase/hydrolase is mainly involved in cell wall reinforcement [52]. The KEGG analysis showed that the gene expression abundances involved in cutin, suberin, and wax biosynthesis were significantly increased, which resulted in cutin, suberin, and wax deposition on the cell walls of stigma cells, thereby increasing cell wall strength [53]. Significant up-regulation of key genes involved in phenylpropanoid biosynthesis further confirmed the accumulation of lignin in stigma the cell wall of water lily after pollination because suberin biosynthesis is closely related to phenylpropanoid biosynthesis [54]. We infer that the accumulation of cutin, suberin, and wax on the cell wall enhances the strength and thickness of the cell wall, thus hindering the pollen germination on the stigma. Therefore, cell wall organization or biogenesis is closely related to low pollen-pistil compatibility of *Nymphaea* species.

### Other enzymes involved in low pollen-pistil compatibility of *Nymphaea* spp

In this study, the combined analysis of transcriptome and proteome showed that the activity of peroxidase increased significantly after pollination. Peroxidase mainly removes peroxides and participates in stress response, auxin metabolism, and signal transduction [27]. Previous research has shown that the peroxidase activity of a mature stigma increased significantly and reached the highest value when the stigma developed was the most receptive to pollen, which is a common method used to judge stigma maturity in the field [49]. Mc Innis was the first to discover stigma-specific peroxidase, which has cell specificity and specific expression patterns, and is specifically expressed in the cytoplasm and cell surface of stigma epidermal cells [27]. Based on our results and previously published papers, we infer that in the process of interaction between the pollen and stigma of *Nymphaea*, peroxidase may directly participate in the process of mutual recognition between the stigma and pollen, perhaps guiding the pollen tube.

Glutathione S-transferase (GST) is a ubiquitous enzyme that regulates plant secondary metabolism, detoxification, and defense, and plays an important role in cell response to stress [55]. A previous study on maize showed that the expression of GST was up-regulated at the early stage of silk-pollen interaction and pollen tube germination [56]. GST was also upregulated in this study. It is possible that GST participates in the pollen-stigma interaction of *Nymphaea*, but its mechanism of involvement needs further study.

Cytochrome P450 has a wide range of catalytic activities. It mainly participates in the synthesis and metabolism of terpenoids, alkaloids, sterols, fatty acids, plant hormones, signal molecules, phenylpropane, flavonoids, and isoflavones [57]. Our results showed that the expression of cytochrome P450 increased significantly. We deduce that cytochrome P450 is involved in the processes of phenylpropanoid and flavonoid biosynthesis, and indirectly participates in the pollen-stigma interaction of water lily.

Mitogen-activated protein kinase (MAPK) is an evolutionarily conserved serine/threonine protein kinase in eukaryotic organisms. It is responsible for regulating signal transduction between cells and amplifying stimulus signals from outside cells to induce appropriate physiological and biochemical reactions in receptor cells [58]. In this study, we found that the expression of MAPK on the stigma of water lily was significantly increased after pollination. We infer that MAPK plays a signaling role in the pollen-stigma interaction and plays an important role in the regulation of the complex network of low pollen-pistil compatibility in water lily.

## Conclusions

In this paper, the differences of stigma transcripts and proteomes at 0, 2, and 6 h after pollination were compared, resulting in the identification of some regulatory genes and functional proteins that may cause pre-fertilization barriers in water lily. The functional analysis showed that differential transcripts were mainly involved in cell wall organization or biogenesis, SAM, hydrogen peroxide decomposition and metabolism, ROS metabolism, secondary metabolism, secondary metabolite biosynthesis, and phenylpropanoid biosynthesis. Our transcriptomic and proteomic analysis highlighted specific genes, incuding those in ROS metabolism, biosynthesis of flavonoids, SAM metabolism, cell wall organization or biogenesis and phenylpropanoid biosynthesis that warrant further study in investigations of the pollen-stigma interaction of water lily. This study strengthens our understanding of the mechanism of low pollen-pistil compatibility in *Nymphaea* at the molecular level, and provides a theoretical basis for overcoming the pre-fertilization barriers in *Nymphaea* in the future.

## Methods

### Experimental materials

An interspecific cross between the female *N*. ‘Peter Slocum’ and male *N. micrantha* was performed as described in a previous report [3]. Self-pollinated ‘Peter Slocum’ and *N. micrantha* were successful. In addition, ‘Peter Slocum’ has a chromosome number of 84 (2n = 6x), whereas *N. micrantha* has a chromosome number of 56 (2n = 4x) [3]. These plants were grown in ponds in Xingxiang, Zhenjiang, Jiangsu Province, China. These plants were collected from shanghai chenshan plant science research center, Chinese Academy of Sciences (Shanghai, China).

The stigmas of 0, 2, and 6 HAP were collected. Each treatment had three biological repeats. The stigmas of 0 HAP (fresh male parent pollen was placed on stigma) were used as the control, and the stigmas from 2 and 6 HAP were used as the treatment to dynamically study the interaction between the pollen and stigma after pollination. After collection, the three samples, the stigmas of 0 HAP, the stigmas of 2 HAP, and the stigmas of 6 HAP, were immediately frozen in liquid nitrogen and stored at − 80 °C.

### High-throughput RNA-seq and data processing

Total RNA was extracted using Trizol reagent according to the manufacturer’s protocol (Takara Bio Inc., Otsu, Japan). The total RNA was checked for quality and quantity using an Agilent 2100 bioanalyzer (Agilent Technologies, CA, USA). The mRNA samples were enriched by oligo (dT) magnetic beads and then cut into fragments with fragmentation buffer at 80 °C. The first strand of cDNA was synthesized with random hexamer as primer. RNase H, dNTPs and DNA polymerase I were used to synthesize the second strand of cDNA using the first strand as template. After purification and end repair, adaptor sequences and double-stranded DNA poly A were attached to the ends of cDNAs. After selecting the fragment size and using Agilent 2100 biological analyzer for quality detection, the cDNA library was constructed by PCR amplification. Finally, the Illumina HiSeq 2500 system was used to sequence the qualified cDNA libraries. Three biological replicates were used in the RNA-seq experiments involving each sample, and sequenced as separate libraries.

Raw data (raw reads) of fasta format were firstly filtered using fastp software. In this step, clean data (clean reads) were obtained by removing reads containing adapter, reads containing ploy-N and low quality reads from raw data. At the same time, Q20, Q30 and GC content the clean data were calculated. All the downstream analyses were based on the clean data with high quality. The transcripts were assembled by Trinity (http://trinityrnaseq.github.io). Clean reads with a certain overlap length were initially merged into long fragments without N (called contigs). TGICL software was used to cluster the related contigs to obtain unigenes (without N) which could not be extended at both ends, and nonredundant unigenes were acquired after removing redundancies [59]. The functional annotation of all-unigenes was performed using a BLAST search (http://blast.ncbi.nlm.nih.gov/Blast.cgi) against the GO, Pfam, KEGG, Nr, and Swiss-Prot databases.

The unigenes obtained using Trinity were used as reference sequences, and the clean reads of each sample were compared to the reference sequences by using the default parameters of bowtie 2 in RSEM software. The results of bowtie2 comparison were statistically analyzed by RSEM, and the number of read counts of each sample compared to each gene was further obtained, and the number of read counts was converted to FPKM, then the gene expression level was analyzed. The FPKM (fragments per kilobase per million mapped reads) method eliminated the effect of gene length and sequencing depth on gene expression calculation. The difference of gene expression between samples was directly compared with FPKM values. The “base mean” value for DEGs identification was obtained by using the DESeq package. The absolute value of log_2_ratio ≥ 1 and FDR ≤0.01 were used as the threshold of significant difference in gene expression between the two samples.

Functional annotation and classification of the DEGs were conducted using the Blast 2 GO program (https://www.blast2go.com/) [60]. Additionally, a KEGG pathway analysis (https://www.genome.jp/kegg/) was performed. The heat map was produced using Cluster 3.0 and treeview.

### Label free analysis of the stigma proteome of water lily

#### Protein extraction and peptide enzymolysis

Protein extraction was performed using the SDT (4% SDS, 100 mM DTT, 150 mM Tris-HCl pH 8.0) method. The protein concentrations were quantified with the BCA Protein Assay Kit (Bio-Rad, USA), and the samples were stored at − 80 °C. Protein digestion (200 μg for each sample) was performed using the filter aided proteome preparation procedure described by Wisniewski [61]. The peptides from each sample were desalted on C18 cartridges, concentrated by vacuum centrifugation, and reconstituted in 40 μL of 0.1% (v/v) formic acid.

#### MS/MS protein identification and quantification

**N**anoLC-MS/MS analysis was performed on each peptide fraction. We first loaded the peptide mixture onto reversed-phase trap column (Thermo Fisher Scientific Acclaim PepMap100, 100 μm × 2 cm, nanoViper C18) and connected it to C18-reversed phase analytical column (Thermo Fisher Scientific Easy Column, 75 μm inner diameter, 10 cm long, 3 μm, C18-A2) in buffer A (0.1% formic acid), and then separated it with the linear gradient of buffer B (0.1% formic acid, 84% acetonitrile) under the flow rate of 300 nL / min controlled by intelliFlow technology.

The LC-MS/MS analysis was carried out on the Q Exactive mass spectrometer (Thermo Fisher Scientific) coupled with a Easy nLC (Proxeon Biosystems, now Thermo Fisher Scientific) for 240 min. Mass spectrometer was operated under positive ion mode. The MS data was obtained through the data-dependent top10 method and dynamically selected the most abundant precursor ions from the measurement scan (300–1800 m/z) to obtain higher collision energy dissociation (HCD) fragments. The automatic gain control target, the maximum injection time and dynamic exclusion duration was set to 1e^6^, 50 ms and 60.0 s respectively. Measurement scans were acquired at 70,000 resolution at 200 m/z; HCD spectra resolution was set to 17,500 at 200 m/z, the normalized collision energy was 30 eV, the isolation width was 2 m/z, and the under-fill ratio was 0.1%.

The MS raw files were dealed with using Maxquant1.5.3.17 software for protein identification [62]. In this study, we searched the acquired MS/MS spectra based on the predicted protein databases translated from the above transcriptome databases. The maximum FDR and the minimum peptide length was set to 1% and six amino acids for both peptides and proteins respectively. Other parameters were set as described below: enzyme = trypsin; max missed cleavage = 2; fixed modification: carbamidomethyl (C); peptide mass tolerance = ± 20 ppm; variable modification: oxidation (M), acetyl (protein N-term). Protein quantification was determined by unique peptides and ‘razor’ [62, 63], and label free quantitation calculation was performed [64]. For each fraction, the peptide was matched in different LC-MS/MS operations based on the mass and retention time (setting matching options between runs in MaxQuant) using a 2 min time window.

DEPs were analyzed as significantly up-regulated or down-regulated. For quantitative changes, a critical value of 2.0-fold was set to determine the up-regulated and down-regulated proteins, at least two replicates with a *p*-value of <0.05.

#### Bioinformatics analysis

Each sample was repeated three times in label free analysis. In order to study the effect of differentially expressed proteins on different biological processes, we carried out enrichment analysis of GO and KEGG. Go and KEGG were enriched by Fisher’s exact test, and the whole quantitative protein annotation was used as the background data set. Benjamini-Hochberg was modified by multiple testing, so that the derived *p*-values could be adjusted [65]. Only pathways and functional classifications with p-values <0.05 were identified as significant.

### Correlation analysis of transcriptomics and proteomics

In a comparison group, if a gene and its corresponding protein were expressed, it is considered that the gene and its corresponding protein were correlated. Next, we determined the significant expression of correlated genes and proteins in the comparison group. If a gene and its corresponding protein were significantly expressed in a comparison group, they were named cor-DEGs-DEPs. GO and KEGG enrichment analysis for cor-DEGs-DEPs were then conducted, and the Spearman correlation coefficient was calculated.

### PRM analysis

#### Selection of target peptides for PRM analysis

Peptide mixture of the nine samples analyzed by label free analysis was prepared by trypsin. The equivalent peptides in each sample was collected, and 2 μg of the collected sample was introduced into the HPLC system through a capture column (5 μm-C18, 100 μm × 50 mm) and an analysis column (3 μm-C18, 75 μm × 200 mm). Next, the separated peptides were analyzed by Q-Exactive mass spectrometer (Thermo Fisher Scientific). Maxquant 1.5.3.17 software was used to analyze the raw files (missed cleavage = 0, enzyme = trypsin/P). The peptide with a score of more than 40 was considered to be the target peptide.

#### Quantitative PRM analysis for target proteins

We selected eight target peptides from the four DEPs for PRM quantitative analysis. We added the peptide retention time calibration mixture to the peptide mixture and used the labeled peptide “TASEFDSAIAQD**K**” (bold “K” for heavy isotope labeling) as the internal standard. We first separated 2 μg of peptide mixture with 20 fmol labeled peptides by HPLC, and then analyzed them by Q-Exactive mass spectrometer. Three times of repeated quantitative analysis were carried out, and raw data was calculated with skyline 3.5.0 software.

## Supplementary information

**Additional file 1.** Differential expression genes in 0, 2 and 6 HAP. (XLS 3738 kb)

**Additional file 2.** Identifiers of proteins contained in the protein group. They are sortted by number of identified peptides in descending order.

**Additional file 3.** Quantitative and differential analysis of protein.

**Additional file 4.** The correlation between DEPs and DEGs.

**Additional file 5.** The cor-DEGs-DEPs genes with the same or opposite trend.

**Additional file 6.** GO enrichment analysis of the cor-DEGs-DEPs genes.

**Additional file 7.** KEGG enrichment analysis of the cor-DEGs-DEPs genes.

**Additional file 8.** Detailed protein quantitative information and significant difference analysis results.

## Data Availability

The raw data from the three samples have been submitted separately to the National Center for Biotechnology Information (NCBI) under the accession number PRJNA548276.

## Abbreviations

cor-DEGs-DEPs: Correlation-DEGs-DEPs
DEGs: Differentially expressed genes
DEPs: Differentially expressed proteins
FPKM: Fragments Per Kilobase Million
GO: Gene Ontology
GST: Glutathione S-transferase
HAP: Hours after pollination
KEGG: Kyoto Encyclopedia of Genes and Genomes
LC-PRM/MS: Liquid Chromatography-Parallel Reaction Monitoring/Mass Spectrometry
MAPK: Mitogen-activated protein kinase
NDEGs: No significant difference in genes
NDEPs: No significant difference in proteins expression
NO: Nitric Oxide
PRM: Parallel reaction monitoring
RNA-seq: RNA sequencing
ROS: Reactive oxygen species
SAM: S-adenosylmethionine metabolism
